# Supplementary figures and images for: Prognostic Significance of Organ-Specific Metastases in Patients with Metastatic Upper Tract Urothelial Carcinoma
Source: J Clin Med. 2022 Sep 9;11(18):5310. doi: 10.3390/jcm11185310 (PMC9504073; doi:10.3390/jcm11185310)

**Supplementary Figure S1. Flowchart of patients included**

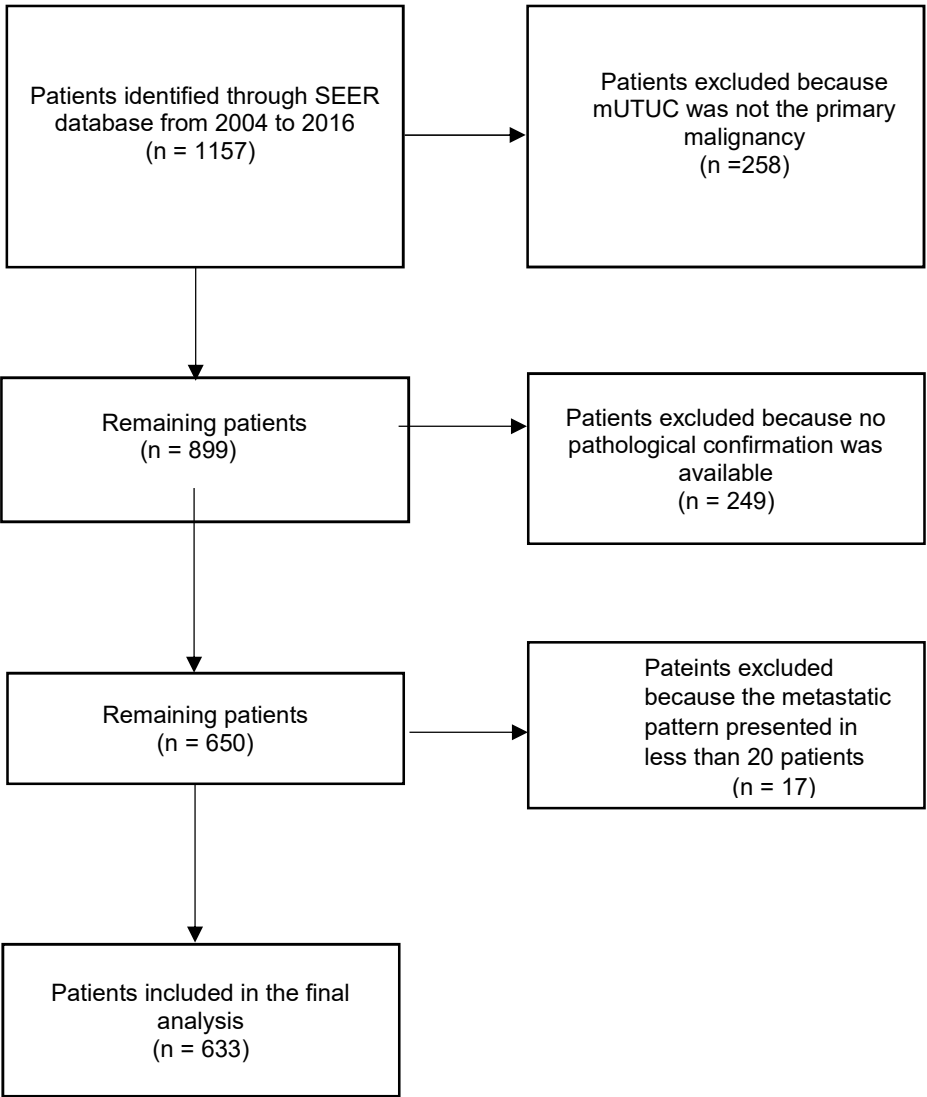

Supplement: Supplementary file 1 [file jcm-11-05310-s001.zip › jcm-1904756-supplementary Figure S1.pdf]
